# Food taboos and their perceived reasons among pregnant women in Ethiopia: a systematic review, 2022

**DOI:** 10.1186/s12884-023-05437-4

**Published:** 2023-02-16

**Authors:** Hadush Gebregziabher, Amaha Kahsay, Fereweini Gebrearegay, Kidanemaryam Berhe, Alem Gebremariam, Gebretsadkan Gebremedhin Gebretsadik

**Affiliations:** 1 Department of Nutrition and Dietetics School of Public Health, College of Health Sciences, Mekelle University, Tigray, Ethiopia; 2 Department of Public Health, College of Medicine and Health Science, Adigrat University, Tigray, Ethiopia

**Keywords:** Food taboos, Perceived reasons, Pregnancy, Ethiopia

## Abstract

**Background:**

There are foods considered as taboo across different communities in the world and in Ethiopia in particular. Although food taboos exist across all ages or physiologic states, they are predominant among pregnant women and children. Identifying such foods among pregnant women is crucial in providing focused interventions and prevents their negative consequences. Therefore, the aim of this review was to review the available evidence on food taboos and their perceived reasons among pregnant women in Ethiopia to provide comprehensive and precise evidence for decision making.

**Methods:**

Electronic search of the literature was made from Pub-Med, Google Scholar, Google Scopus, and Medline databases using search terms set based on the PICO/PS (Population, Intervention/exposure, Comparison, and Outcome) and PS (Population and Situation) search table. The search was made from December 05, 2020 – December, 29, 2021, and updated on January, 2022. All quantitative and qualitative studies published in English were included in the review. The systematic review protocol was registered at INPLASY (Registration number: INPLASY202310078). The outcome of interest was food taboo for pregnant women and its perceived reasons. The results of the review was narrated.

**Results:**

After identifying eighty two articles, thirteen were found eligible for the review. Vegetables, fruits, and fatty foods like meat, and dairy products were considered as taboo for pregnant women in different parts of Ethiopia. The reasons stated for the food taboo vary from fear of having a big baby, obstructed labour, and abortion to evil eye and physical and aesthetic deformities in the newborn.

**Conclusions:**

Though not uniform across the country, there are foods considered as taboo for pregnant women in Ethiopia due to several perceived reasons, misconceptions, and societal influences. This could increase the risk of malnutrition and could have short and long term consequences on both the mother and her growing foetus. Therefore, context specific nutritional counseling with emphasis during ante-natal care and post-natal service is important.

## Background

Pregnancy is a particular period when physiological nutrient demands are substantially increased. Maternal nutrition guidelines recommend pregnant women to meet this increasing amount and quality of nutrient requirements [1]. A healthy maternal diet during pregnancy contains adequate energy, fats, proteins, vitamins and minerals, obtained from consuming a variety of food groups including whole grains, vegetables, fruits, legumes, milk, meat, fish, and nuts [2]. However, in many societies, there are foods considered as taboo for pregnant women. This compromises the ability of pregnant women to meet the recommended dietary intake. It further puts the mother and her newborn at a greater risk of adverse outcomes [3].

In low and middle income countries like Ethiopia where girls and women usually have unjust access to basic entities like health care and education, maternal undernutrition remains a major concern. Pregnant women in these countries usually have insufficient food intake especially late in the 2nd and 3rd trimesters resulting in poor nutritional status of the mother and her growing fetus [4]. While dietary intake during pregnancy is affected by several factors including affordability and accessibility of food, restricted eating of some food items because of cultural prohibitions, has also been a prominently reported attribute [5, 6].

The extent of the practice of food taboos and the specific tabooed foods vary from one community to another. However, compared to urban and more educated communities, food taboos are generally more common among rural and less educated ones [7]. Food taboo related information is mainly transferred from people considered highly influential including grandmothers, elders, and experienced mothers. Such highly rated and respected members of a community play central roles in encouraging the public to practice food taboos by spreading information on which specific foods are taboos and why [8–10].

Undernutrition among pregnant women has been one of the serious public health challenges in Ethiopia. This could be related to the high magnitude of maternal and child mortality in the country [11]. A study conducted in Western Ethiopia showed that nearly 30% of pregnant women are undernourished (mid upper arm circumference less than or equal to 21 cm) [12]. Food taboos prevent eating certain food items thus compromising one’s dietary diversity and quality which, in turn, would lead to poor health and nutritional outcomes [13, 14]. Evidence shows that food taboos are largely associated with maternal and fetal malnutrition during pregnancy [15, 16] and could have consequences on the mothers and their children later in life [17].

The risk of undernutrition during pregnancy is enhanced due to the physiological increases in nutrient demand and the subsequent incapability to meet this demand by women [18]. This situation often even gets worse due to the food taboo-related limited dietary intake [19] increasing the likelihood of developing scores of negative pregnancy outcomes, maternal anemia, and low birth weight (LBW) [20]. In Ethiopia, about 13.5% of newborns are LBW (birth weight less than 2500 g) [21]. Such problems during pregnancy could also impose deleterious effects on child survival and economic productivity later in adult life.

Though it is not uniform, the practice of food taboos is a common problem in developing countries. Based on the few studies available in Ethiopia, food taboos prevail with varying type and severity across the different culture and topography of the country. For instance, in Oromia region linseed, honey, and milk/ yoghurt are commonly avoided food items for a perceived fear that these food items would be plastered on the fetal head [22]. Besides, while organ meat and dark green leafy vegetables were avoided for fear of infection in Addis Ababa [23], consumption of livestock derived foods was restricted in South nation, nationalities, and people’s region (SNNPR) for fear of difficult delivery that could lead to increasing size of the fetus [24].

To our knowledge, there is paucity of systematically narrated evidence on food taboos and their perceived reasons among pregnant women in Ethiopia. This review is intended to synthesize information on the main foods considered taboos by pregnant women and their perceived reasons using a systematic search of the available literatures in Ethiopia. This would help to design and implement evidence based interventions.

## Methods

### Searching strategy

A comprehensive search was made from Pub-Med, Google Scholar, Google Scopus, and Medline databases. The search was done using search terms including “maternal dietary practice”, OR “harmful traditional practice on feeding” and “food taboos for pregnant women”, “Impact of food taboo” OR “feeding practice”. These search terms were set using search tables of the PICO (Population, Intervention/Exposure, Comparison, and Outcome) and PS (Population and Situation) for quantitative and qualitative articles, respectively. Besides, reference lists of this systematic review included articles and reviews were also scanned for potential articles. The search was made from December 05, 2020 – December 29, 2021, and updated on January, 2022. All quantitative and qualitative studies published in English were included in the review.

### Study selection and data extraction

Studies were identified by searching electronic databases, scanning reference lists of articles, grey literature, and other non-bibliographic sources. Two authors performed the search activities independently. The following information was extracted from each study that met the inclusion criteria: the name of the first author, year of publication, study design, and food taboos and their perceived reasons. The first screening was based on a double-screening of titles and abstracts. Results which met explicit exclusion criteria were excluded. In the second step, the remaining articles were assessed for full-text reading. In case of disagreement among reviewers, a third reviewer assessed the study and a decision for inclusion was reached by consensus. The mean age of the participants and the educational status were extracted from the articles.

### Eligibility criteria

Inclusion criteria: all quantitative and qualitative primary research articles published related to food taboos, food prohibitions, and restrictions during pregnancy and perceived reasons. Studies which were not in line with our objectives in terms of abstract, full text content, and duplicated articles were excluded.

**Fig. 1:**
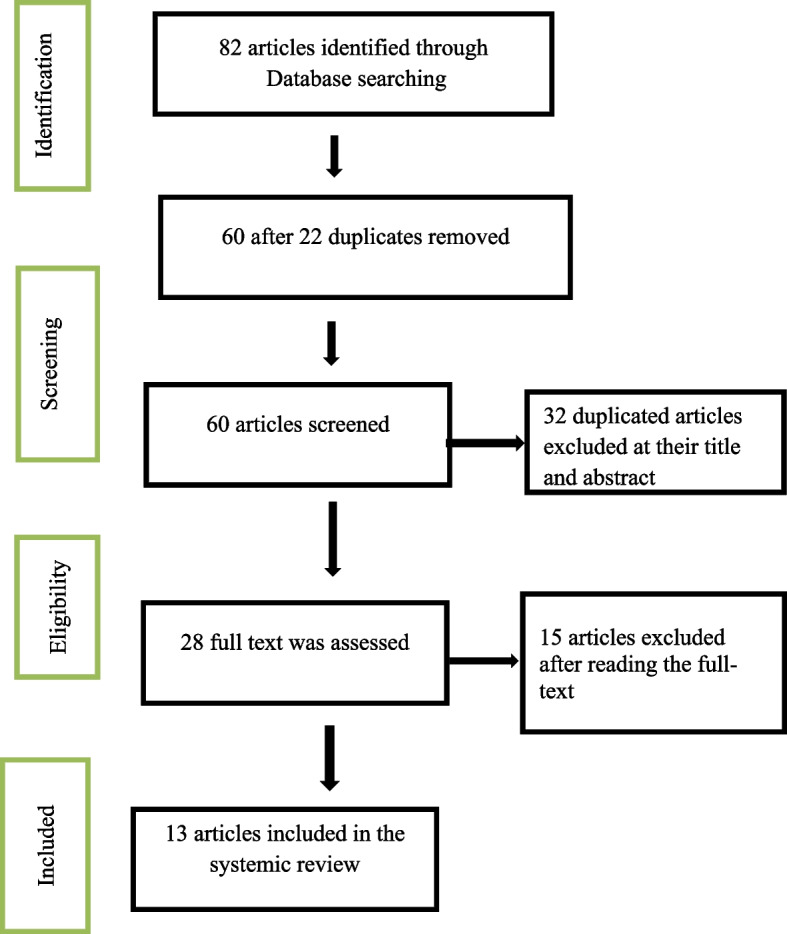
Flow chart showing the selection of articles included in the systematic review in Ethiopia, 2022

During the primary search, 82 records were identified. After screening the title and abstract of the studies, 22 records were excluded leaving 60 records. Again, we excluded 32 duplicate records and only 28 records were left. After assessing the full texts we excluded 15 records using the exclusion criteria. Finally, 13 studies were found to be eligible for this systematic review (Fig. 1). The results of the review was synthesized descriptively and presented under the themes.

### Protocol registry

The systematic review protocol was registered at INPLASY (Registration number: INPLASY202310078).

### Assessment on quality of the studies

The studies were assessed using the criteria proposed check list called the Joanna Briggs institute (JBI) critical appraisal for systematic review tool for quality assessment [25]. The parameters include how the review questions were stated, appropriateness of the inclusion criteria, search strategy, source and resource used to search, criteria for appraising studies, number of authors conducted for appraisal, how errors were minimized in the data, method used to combine studies, assessing publication bias, supporting recommendation for policy /or practice, and use of specific directive approach for new research.

## Results

### Characteristics of the included articles

Thirteen articles fulfilled the inclusion criteria. The included articles were from Addis Ababa, Tigray, Afar, Oromia, SNNPR, and Amahara Regional states (Table 1).

**Table 1:** Characteristics of articles included in a systematic review of food taboos and their perceived reasons among pregnant women in Ethiopia, 2022

S.N	Authors and year	Method	Sample size	Study area
1	Robert Wondimu, Esubalew Tesfahun, Zalalem Kaba 2021 [26]	Mixed	384 & 4 FGDs	Sendafa Beke, Oromia
2	Freweini Gebrearegay, Lemlem Weldegerima Gebremariam, Selemawit Asfaw Beyene 2020 [27]	Quantitative	332	Mekelle, Tigray
3	Mohammed et al., 2019 [23]	Quantitative	592	Addis Ababa
4	Hayelom Abadi Mesele, 2018 [28]	Qualitative	12 IDIs, 15 KIIs, & 3 FGDs	Raya Alamata, Tigray
5	Wollelaw Getnet, Wubie Aycheh, and Taddele Tessema, 2018 [29]	Quantitative	307	Debremarkos, Amhara
6	Afework Mulugeta, Mekonnen Haileselassie, Znabu Hadus, Zewditu Getahun and Alessandra N. Bazzano, 2018 [30]	Qualitative	14 FGDs & 90 KIIs	Rural Tigray
7	Znabu Hadush, Zewdie Birhanu, Mulugeta, 2017 [31]	Qualitative	4 FGDs & 8 KIIs	Abala, Afar
8	Vidanka Vasilevski & Mary Carolan-Olah, 2016 [32]	Mixed	14 articles	Ethiopian migrant pregnant women
9	Taddese Alemu, Zerfu Melaku, Umeta and Kaleab Baye., 2016 [33]	Qualitative	8 FGDs & 38 KIIs	Arsi, Oromia
10	Kuche Desalegn, singah Pragya, Mogus Debebe, 2016 [34]	Quantitative	153	Wondogenet, SNNPR
11	Meresa Gebremedhin, Fentie Ambaw, Eleni Admassu and Haileselassie Berhane, 2015 [35]	Mixed	308 & 3 FGDs	Tigray Region
12	Nejimu Biza Zepro, 2015 [22]	Quantitative	295	Shashemene, Oromia
13	Tsegaye Demissie, Nelson Muroki, Wamboi Kogi-Makau, 1998 [24]	Quantitative	295	Hadiya, SNNPR

*FGD* Focus Group Discussion, *IDI* Indepth Interview, *KII* Key Informant Interview, *SNNPR* South Nations Nationalities, and Peoples’ Region

### Food taboos and their perceived reasons among pregnant women in Ethiopia

According to a study in Shashemene District of Oromia Region, 147 (49.8%) study participants encountered food taboos at least for one food item [22]. This study also showed that eating honey during pregnancy was considered a taboo for perceived reasons that it leads to a painful prolonged false labour and that it is also the main cause of constipation during the course of pregnancy [22]. Besides, according to a study conducted in Addis ababa, linseed, honey, and milk/ yoghurt were found to be restricted during pregnancy for the perception that they could be plastered on the fetal head and result in fatty baby and difficult delivery, fear of abortion, evil eye, and fetal abnormality [23]. This study also described that pregnancy related food taboos were responsible for avoidance of at least one food item for about a fifth (18.2%) of the study participants [23]. Another study conducted in Raya Alamata, Tigray region found that eating brassica seed (locally known as “senafiche”), pepper, sugar cane, milk, cheese, honey, meat, banana, tomato, onion, cactus fruit, chickpea, lentils, and roasted grains (locally called “kollo”) were considered as taboos by women during pregnancy for perceived reasons including prolonged labour, difficulty in delivery, abortion and miscarriage, large fetus, and feeling of indigestion [28].

In a quantitative study from Amhara Region, which reported a 27% practice of food taboos, certain fruits and vegetables such as banana, pimento, cabbage, and sugarcane were considered taboos for perceived reasons including that banana attaches to the head of the fetus, pimento burns the fetus, cabbage disturbs the fetus, and sugarcane increases seminal fluids [29]. A qualitative study done in rural Tigray found that pregnant women avoided eating food items such as yogurt, banana, legumes, honey, and “kollo” (roasted barley and wheat) because these foods were believed to cause abortion, abdominal cramps in the mother and new-born, prolonged labour, or coating of the fetus’s body [30]. Another study in Mekelle city reported that around 12% of the pregnant women avoided at least one type of food during their current pregnancy for one or more reasons [27]. In perception, if a pregnant woman eats leafy vegetables, the leaf passes to the womb and attaches to the baby’s head to form some “particles” that are considered harmful to the child and are even considered to cause immediate death of the new born [33]. In another study, pregnant women reported avoiding dairy products like yoghurt and cheese, particularly as the gestational age advances, because of the perception that dairy products can pass to the womb and attach to the baby’s head [34].

In a study in Abeala, Afar region, bread-like foods locally named as “Burkutta”, “Himbassha”, “Bahamo”, and “Mengelle” and roasted grains (“kolo”) were tabooed for pregnant women for perceived reasons of severe bleeding during delivery and remaining painted at the foetus’s head till birth, respectively [31]. In another study, more than half (55.3%) of the total pregnant women reported food taboo for at least one food item [26]. On the same note, it is perceived that pregnant woman should avoid eating high fat foods like meat, camel milk and yoghurt, which are locally considered as “good foods”, to prevent the foetus from being large [26, 35]. Summaries are depicted below (Table 2).

**Table 2:** Food taboos and their perceived reasons among pregnant women in Ethiopia, 2022

S.N	Sociodemography				Foods considered as taboos for pregnant women	Perceived reasons
	Study area		Mean Age of study participants	Majority educational status		
	Town/region	Rural/Urban				
1	Addis Ababa	Urban	25	Grade 1–8	Dark green leafy vegetables (spinach, lettuce, kale, and broccoli)	Fear of infection of the foetus and the mother, especially when eating vegetables
					Raw meet,organ meat and except liver	Large baby and difficult birthing, abortion, offensive vaginal discharge
					Banana	Food sticking’ on the foetus’ head, especially when
2	Raya Alamata, Tigray	Urban	29	Primary school completed	Brassica seed (Senafiche), pepper, sugar cane, milk, cheese, honey, meat, banana, tomato, onion, cactus fruit, chickpea (Shimbera), lentil (misir), roasted grains (kollo)	Fear of difficulty in delivery, fear of prolonged and pain Labour, abortion and miscarriage, large fetus, and feeling of indigestion
					Milk and milk products	Milk and milk products stick on the head and face of the fetus. This could spoil the face of the foetus and result in delivering a child with ugly face
3	Awabe, Amhara	Rural	25	Unable to read and write	Fruit and vegetables including banana, pimento, cabbage and sugarcane	Banana could be attached to the head of the fetus, pimento burns the fetus, cabbage disturbs the fetus, and sugarcane increases seminal fluids
					Cereals like wheat and oilseeds like linseed, nigerseed, groundnut and salty diets	Increase the weight of the fetus, making it difficult to deliver. Linseed causes loss of strength in the fetus, and nigerseed makes the color of the fetus black
					Drinks like coffee, tea, Coca-Cola, and porridge	Drinks like coffee, tea, coca, and porridge burn the foetus causing abnormality and coca cola drink causes abortion
4	Mekelle, Tigray	Urban	28.5	Seconday education	Legumes, mustard, porridge, bananas, and whole grains in the form of ‘‘kollo”, honey, and milk products (yogurt and milk)	Legumes (beans and chickpeas) are believed to cause abdominal cramps in both mother and fetus, prolong labor, and cause abortion. Whole grains in the form of “kollo” were believed to exacerbate labor pain, and cause postpartum abdominal cramps, heartburn, and nausea in the mother
					Porridge, bananas, and milk products	Become coated to the body of the fetus and make the baby very big, causing difficult/prolonged labor
					Honey	Causes abortion, and exacerbates labor pain
5	SendafaBeke, Oromiya	Rural	27	Elementary education	Linseed	Causes abortion
					Eating leafy vegetables	Leafy vegetables could go to the womb and attach to the baby's head causing particles to appear on the fetal head at delivery
					Egg, milk and milk products	Could make the foetus too big making it very dangerous to the life of the mother and the child during labor
6	Rural Tigray	Rural	30	Unable to read and write	Kollo (roasted) of chickpea and wheat	Causes abdominal cramp in newborns
					Hot coffee	Causes balding in children
					Green pepper and “senaficho” (dressing made from brassica)	Green pepper affects the eye of the foetus and “Senaficho” could cause miscarriage
					Alcohol	Affects the health of the baby
					Cereals like maize and millet	Maize causes nausea and millet could lead to constipation
					Nigerseed oil	Causes skin darkness (black color)
					“Shiro” (stew/sauce made of legumes), pea, bean	“Shiro” provides no calories and do not prevent from anemia. Peas and beans cause nausea during pregnancy
7	Arsi,Oromiya	Rural	31	Elementary education	Leafy vegetables like cabbage	Form “particles” that are harmful to the child and are even likely to cause immediate death to the newborn
					Dairy products like milk, yogurt, and cheese as gestational age advances	Dairy products can pass to the womb and attach to the baby’s head
8	Ethiopian migrant pregnant women	-	-	-	Linseed, honey, milk, and nuts	Fear of giving birth to a ‘fatty baby’, having a baby with discolored skin, abortion and stillbirth
9	Wondogenet, SNNPR	Urban	25	No formal education	Legumes	Because of like and dislike
10	Abeala, Afar	Rural	28	Unable to read and write to elementary	Bread-like local foods like “Burkutta”, “Ambassha”, “Bahamo” and “Mengelle”	Bleeding during labour and delivery could occur
					Roasted grains (“Kalo”)	Could be painted at the head of the baby
					High-fat foods like, meat, camel milk, and yoghurt	Could prevent the foetus from being large
11	Urban Tigray	Urban	26.9	Elementary education completed	Meat, fat, and butter	Could enable the foetus to grow larger beyond the birth canal resulting in severe complications during birth
					Honey	Could lead to a prolonged painful false labour and may cause constipation
					Eggs, meat, and much cow’s milk	Could cause unnecessary growth of the foetus
12	Shashemene, Oromiya	Urban	27.5	Unable to read and write	Linseed, honey and Milk/ yoghurt	Could be plastered on the fetal head, make fatty baby and difficult delivery, fear of abortion, evil eye, and/or fetal abnormality
13	Hadya, SNNPR	Urban	25.2	Elementary education	Milk and cheese, linseed, fatty meat and banana	Difficult delivery as the result of increased size of the fetus

## Discussion

This review revealed that there are foods considered as taboos among pregnant women for several perceived reasons and that such restrictions are higher during the last trimester of pregnancy. Foods considered as taboos among pregnant women in Ethiopia include vegetables, solid foods made of cereals, dairy products, meat, and oil seeds like linseed. The main perceived reasons for such practices were the effect of these foods on increasing the size of the foetus which they supposed could later cause complicated labour and negative birth outcomes and cosmetic effect of some of the foods on the newborn. Similarly, a study from Egypt reported that carbohydrate based food groups were avoided by pregnant women for perceived reasons that they could cause bloating and excessive weight gain in the mother [36]. Avoidance of these foods may negatively affect the dietary intakes of these women, as dietary diversity recommendations for pregnant women emphasize the need for pregnant women to eat diverse foods with adequate energy, protein, fat, fiber, and micronutrients [37].

This review has seen some variation in the meaning given to the same food items in differet locations that are considered taboos during pregnancy. This could be due to differences in cultural values and knowledge and experience of highly influential and experienced community figures like grandmothers, elders, or others who spread food taboo related information.

This review showed that pregnant women should avoid eating high fat foods such as meat and milk to prevent from having big foetus that could later lead to difficult labour. This is consistent with a study conducted in Kenya which stated that pregnant women do not usually eat meat for fear of having obstracted labour [38]. Such taboos related to animal source of foods (ASF) might lead to poor pregnancy weight gain and increase the risk of giving birth to a low birth weight baby. Moreover, low consumption of ASFs during pregnancy could also lead to protein, energy, and micronutrient deficiencies, as these foods are also good sources of many bioavailable micronutrients [39].

The fear of having big baby and difficult labour, abortion, placental disruption was documented in studies conducted in Aligarh, India, in which pregnant women were supposed to avoid papaya, fish, badi food (which cause gas in stomach), citrus foods, groundnuts, and tea [40] and in Ghana in which eggs, fresh meat, fresh mik, and cold and sugary foods were considered taboos for pregnant women [41]. Another study done in India also reported taboos during pregnancy on sugarcane juice, hot foods, carbonated drinks, tapai or fermented glutinous rice, bamboo shoots, and cold foods for perceived reasons including risk of abortion, excessive bleeding during labour, and deformities in the newborn [42]. Moreover, a study in Tajikistan described that consumption of carbohydrates during pregnancy leads to excessive weight gain and a risky delivery because high gestational weight gain “makes the baby very big” [43]. A study among malay pregnant women found out a significant association between practice of food taboos and weekly rates of weight gain [44].

However, continued exclusion of carbohydrates from the prenatal diet can contribute to maternal under nutrition, which holds additional implications for child health as the primary cause of LBW. The recommended dietary allowance for carbohydrate intake for pregnant women is 175 g per day, which is 45 g more than the recommended dietary allowance for non-pregnant [45].

Pregnant women are recommended to take extra nutrients than other women physiologically due to the increased basal metabolic rate by 10 – 15 percent and energy needed, especially from 20 weeks later, for the growing foetus and for the placenta [46]. The raised energy requirements are maintained by foods containing carbohydrate, protein, fat, vitamins, and minerals by adding one extra meal on the normal meal sequence. However, this review revealed that some pregnant women are restricted from consuming vegetables, grains, ASFs and fiber rich foods due to the stipulated taboos in the community. This could lead the mother to burn its own fat and then its tissue proteins which further could lead to energy deficiency. Besides, restricting fiber rich foods like linseeds and legumes could lead to constipation and other disturbances of the colon. Moreover, denying ASFs like milk and meat during pregnancy could result in retardation of growth of the foetus and its future wellbeing. Therefore, the role of food taboos could be of paramount importance in the challenges faced during the prevention of malnutrition in the first one thousand days of life. This implies the need for unreserved effort for the implementation of nutritional social behavioral change and communication strategies to mitigate the practice of food taboos and their consequences during pregnancy.

The systematic review’s methodological quality and its efforts in dealing with possible bias in its design and/or analysis was assessed and deemed appropriate using the JBI critical appraisal tool [25]. The review question was clearly and explicitly stated as food taboos and their perceived reasons among pregnant women in Ethiopia. The inclusion criteria were appropriate in a way they match the review question. Articles were identified appropriately using a comprehensive electronic search strategy using relevant terms, sources, and resources. Critical appraisal of the review was conducted by two independent reviewers. To minimize bias in data extraction, training was provided to the authors and searching and data extraction were made two times by separate individuals. Methods used to synthesize results are in harmony with the methodology used. Besides, the findings were supported with clear summary descriptions and explanations taken from the original articles. The findings of the review are able to lead to policy recommendations. This review has recommended researchers to expand the geographical scope of the study and to work on behavioral change interventions to mitigate food taboos and their effects on maternal and child nutrition.

### Strengths and limitations of the review

This review had its strengths and limitations. Its main strength is the fact that it used clearly specified inclusion and exclusion criteria and a comprehensive search strategy to minimize publication bias. The most notable limitation of this review is that it couldn’t present numbered outcomes or effects because it didn’t conduct a meta-analysis of the studies. Besides, the articles included in this systematic review do not represent all regions of Ethiopia.

## Conclusions

This review found out that, in Ethiopia, food taboos among pregnant women exist with varying features and for several perceived reasons. Such practices are leading pregnant women in Ethiopia to miss foods which provide critical nutrients for themselves and for the growth of their foetus. As a solution to deal with these practices, studies set out that guided context specific nutritional counseling with emphasis during ante-natal care and post-natal service on the dietary practices of pregnant women is an effective approach. Moreover, conducting community based interventional studies could help in providing targeted and specific nutritional interventions in the country.

## Data Availability

All data regarding this systematic review are contained and presented in this document.
